# Risk for Suicide Attempts Assessed Using the Patient Health Questionnaire–9 Modified for Teens

**DOI:** 10.1001/jamanetworkopen.2024.38144

**Published:** 2024-10-08

**Authors:** Fuchiang Tsui, Victor M. Ruiz, Neal D. Ryan, Lingyun Shi, Nadine M. Melhem, Jami F. Young, Molly Davis, Robert Gibbons, David A. Brent

**Affiliations:** 1 Tsui Laboratory, Department of Biomedical and Health Informatics, Children’s Hospital of Philadelphia, Philadelphia, Pennsylvania; 2 Department of Anesthesiology and Critical Care, University of Pennsylvania, Philadelphia; 3 Department of Biostatistics, Epidemiology, and Informatics, University of Pennsylvania, Philadelphia; 4 Department of Psychiatry, University of Pittsburgh School of Medicine, Pittsburgh, Pennsylvania; 5 Department of Child and Adolescent Psychiatry and Behavioral Sciences, Children’s Hospital of Philadelphia, Philadelphia, Pennsylvania; 6 Department of Psychiatry, University of Pennsylvania Perelman School of Medicine, Philadelphia; 7 Department of Medicine and Public Health Sciences, University of Chicago

## Abstract

**Importance:**

Suicide is a leading cause of death in US youths.

**Objective:**

To assess whether screening with supplemental items 10 to 13 on the Patient Health Questionnaire–9 modified for teens (PHQ-9M) improves prediction of youth suicide attempts beyond the information provided by the first 9 items alone (the PHQ-9).

**Design, Setting, and Participants:**

This retrospective cohort study used a retrospective cohort of adolescents aged 12 to 17 years who were screened for depression in outpatient facilities within a pediatric health care system between January 1, 2016, and December 31, 2022, with up to 1 year of follow-up to assess the occurrence of suicidal behavior. Follow-up was completed on December 31, 2023.

**Exposure:**

Screening with the PHQ-9M.

**Main Outcomes and Measures:**

This study developed and compared prediction using 3 Cox proportional hazards regression models (CR-9, CR-13, and CR-3) of subsequent suicide attempts, determined by the hospital’s electronic health records up to 1 year following the last PHQ-9M screening. The CR-9 model used the PHQ-9 and the CR-13 model used all 13 items of PHQ-9M. The CR-3 model used the 3 most impactful variables selected from the 13 PHQ-9M items and PHQ-9 total score. All models were evaluated across 4 prediction horizons (30, 90, 180, and 365 days) following PHQ-9M screenings. Evaluation metrics were the area under the receiver operating characteristic curve (AUROC) and the area under the precision recall curve (AUPRC).

**Results:**

Of 130 028 outpatients (65 520 [50.4%] male) with 272 402 PHQ-9M screenings, 549 (0.4%) had subsequent suicide attempts within 1 year following the PHQ-9M screening. The AUROC of the CR-9 model in the 365-day horizon was 0.77 (95% CI, 0.75-0.79); of the CR-13 model, 0.80 (95% CI, 0.78-0.82); and of the CR-3 model, 0.79 (95% CI, 0.76-0.81); the AUPRC of the CR-9 model was 0.02 (95% CI, 0.02-0.03); of the CR-13 model, 0.03 (95% CI, 0.02-0.03); and of the CR-3 model, 0.02 (95% CI, 0.02-0.03). The 3 most impactful items using adjusted hazard ratios were supplemental item 13 (lifetime suicide attempts; 3.06 [95% CI, 2.47-3.80]), supplemental item 10 (depressed mood severity in the past year; 2.99 [95% CI, 2.32-3.86]), and supplemental item 12 (serious suicidal ideation in the past month; 1.63 [95% CI, 1.25-2.12]). All of the models achieved higher AUROCs as prediction horizons shortened.

**Conclusions and Relevance:**

In this cohort study of adolescent PHQ-9M screenings, the supplemental items on PHQ-9M screening improved prediction of youth suicide attempts compared with screening using the PHQ-9 across all prediction horizons, suggesting that PHQ-9M screening should be considered during outpatient visits to improve prediction of suicide attempts.

## Key Points

**Question:**

Do supplemental items 10 to 13 on the Patient Health Questionnaire–9 modified for teens (PHQ-9M) improve prediction of youth suicide attempts beyond the information provided by the first 9 items (PHQ-9) alone?

**Findings:**

In this cohort study of 130 028 youths screened with the PHQ-9M as outpatients between 2016 and 2022, 549 had documented suicide attempts within 1 year of screening determined by electronic health records. The Cox proportional hazards regression model using the 13-item PHQ-9M outperformed the model using the PHQ-9.

**Meaning:**

This study suggests that PHQ-9M screening should be considered during outpatient visits to improve prediction of suicide attempts.

## Teaser

This cohort study compares performance of the 13-item Patient Health Questionnaire–9 (PHQ-9) modified for teens and the 9-item PHQ-9 for subsequent first suicide attempt within 1 year of screening among US adolescents aged 12 to 17 years.

## Introduction

Suicide was the second-leading cause of death among US adolescents aged 12 to 17 years in 2020.^1^ From 1999 to 2020, suicide mortality in this age group increased from 4.1 to 6.3 per 100 000 population (a 54% increase) in the US.^1^ One strategy for reversing this disturbing trend is screening for depression and suicidal risk in pediatric primary care. Better detection and management of depression in primary care, including adults and adolescents, has been associated with regional reductions in suicide.^2,3,4^

The Patient Health Questionnaire–9 (PHQ-9) is a widely used self-report screen for depression in adults and adolescents during outpatient visits and has been shown to have good sensitivity and specificity for the detection of interview-validated major depression.^5,6^ More recently, it has been recognized that item 9 on the PHQ-9, measuring the frequency of current suicidal ideation or thoughts of self-harm, can identify individuals at risk for suicide attempts and deaths in both adults and adolescents.^7,8,9^ While the risk for suicide attempts and deaths increased with increased frequency of suicidal ideation reported on item 9, more than one-third of those who made suicide attempts or died by suicide within 30 days of screening reported no suicidal ideation.^9^ Similarly, the PHQ-9 used in pediatric primary care screening missed some individuals at suicidal risk who were detected with the addition of the 4-item Ask Suicide Screen Questionnaire^10^ during emergency department visits.

The PHQ-9 modified for teens (PHQ-9M), a self-report screen for depression during pediatric primary care visits, was developed based on the PHQ-9,^5,6^ PHQ for Adolescents,^11^ and the Columbia Depression Scale.^12^ The PHQ-9M retains the 9 core items from PHQ-9 and 4 supplemental items assessing depression in the past year (item 10), functional impairment (item 11), serious suicidal ideation in the past month (item 12), and lifetime history of suicide attempts (item 13).^11^ These supplemental items were aimed at improving detection of suicidal risk and severity of depression. While studies have examined the demographic correlates of suicidal ideation and behavior identified using the PHQ-9M,^13^ there have been limited studies on its predictive validity for adolescent suicide attempts, both alone and compared with the PHQ-9.

In clinical practice, a common approach to identifying youths at risk of suicide uses the suicidal-thought-and-behavior (STB) rule, which is triggered by a positive response to any of the 3 suicide-related items (items 9, 12, or 13) on the PHQ-9M, such as a nonzero score on item 9 or a “yes” response on item 12 or 13.^14^ However, it is unclear whether such practice is optimal for predicting suicide attempts or if there are other combinations of items on the PHQ-9M that may better predict risk; we believe that machine learning can illuminate the optimal subset of variables for prediction of suicidal risk.^15^

A recent review of 67 studies^16^ found that female adolescents had a higher risk of suicide attempts than male adolescents. This outcome was attributed to the fact that female adolescents have greater prevalence of suicide attempts, internalizing symptoms, and psychological abuse.

In the present study, we hypothesized that the PHQ-9M would provide improved clinical utility compared with the PHQ-9 for predicting adolescent suicide attempts.^17^ Throughout this study, we used the first 9 questions from the PHQ-9M to represent the 9 core items of PHQ-9 (ie, excluding the 4 supplemental items in the PHQ-9M). This study aimed to: (1) compare prediction performance of 2 regression models using the 13-item PHQ-9M and 9-item PHQ-9 for a subsequent first suicide attempt within 1 year of screening; (2) examine the multivariate associations between individual items of the PHQ-9M with suicide attempt outcome; (3) develop a parsimonious regression model using a few variables from the PHQ-9M as an alternative to existing risk-screening strategies for suicide attempt; and (4) compare the predictive power among the 3 regression models, common clinical screening strategies, and the PHQ-9 total score (PHQ-9 TS).^16^ To our knowledge, this is the first study comparing clinical utility between PHQ-9M and PHQ-9 for predicting the subsequent first adolescent suicide attempt. Our findings can facilitate additional options for using depression screening data to enhance suicide attempt risk prediction and therefore clinical follow-up.

## Methods

The Institutional Review Board at the Children’s Hospital of Philadelphia (CHOP) reviewed and determined this study to be exempt research. This study follows the guidelines of the Strengthening the Reporting of Observational Studies in Epidemiology (STROBE) and Transparent Reporting of a Multivariable Prediction Model for Individual Prognosis or Diagnosis (TRIPOD).

### Cohort Data

**Figure 1.**
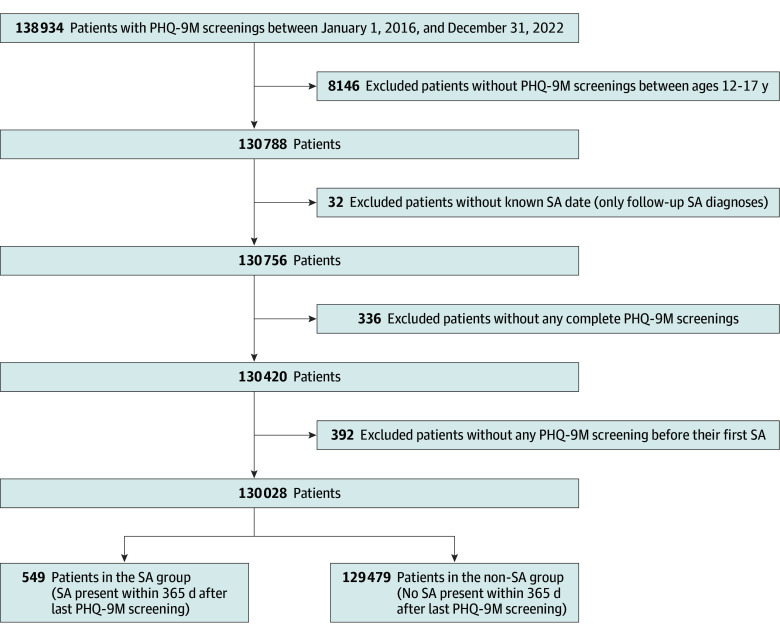
Flowchart of the Cohort Inclusion and Exclusion Process PHQ-9M indicates Patient Health Questionnaire–9 modified for teens; SA, suicide attempt.

We conducted a retrospective cohort study by retrieving historical electronic health record (EHR) data (including self-reported race and ethnicity demographics) among patients aged 12 through 17 years who completed PHQ-9M questionnaires during visits to CHOP outpatient facilities. The study period for patient inclusion spanned from January 1, 2016, to December 31, 2022. The institutional guideline to screen for depression using PHQ-9M during all annual well visits for patients 12 years and older was not initiated until November 2017; prior to that time, the institutional guideline specified that depression screenings should occur at all annual well visits at 16 years of age. The description of PHQ-9M measures in eMethods in Supplement 1 provides further details. Patients who completed PHQ-9M questionnaires during the inclusion period were followed up for 1 year (to December 31, 2023) for their first suicide attempt (the main outcome), identified via the *International Statistical Classification of Diseases, Tenth Revision* (*ICD-10*), codes following the definition by Hedegaard et al.^18^ We excluded *ICD-10* diagnostic codes with D or S suffixes, which denote visits not related to an initial suicide attempt but to follow-up or sequela visits. Additionally, the PHQ-9M screenings after the first suicide attempt during the study period were excluded. Figure 1 shows the study flowchart.

### Statistical Analysis

To evaluate the impact of PHQ-9M with supplemental items 10 to 13, we developed 3 Cox proportional hazards regression (hereinafter referred to as Cox regression) models^19^ using all or partial items on PHQ-9M. We chose the Cox regression over other regression models to consider multiple PHQ-9M screenings a patient might have (ie, treated as item-level plus total score time-varying predictors). First, we compared performance between one model using only the PHQ-9 and the other using the full 13 items of PHQ-9M for subsequent prediction of adolescent suicide attempts within 1 year from the last PHQ-9M screening. Second, we developed a data-driven parsimonious Cox regression model that selected the top 3 variables with the greatest impact from 14 variables: 13 PHQ-9M items and the PHQ-9 TS, followed by outcome prediction comparisons among the 3 regression models, the PHQ-9 TS, and various screening strategies (eg, the STB rule using items 9, 12, and 13). We performed a sensitivity analysis comparing different sizes of parsimonious models. Finally, we examined the hazard ratios (HRs) of the 13 items on PHQ-9M.

#### PHQ-9 Total Score

The PHQ-9 TS was computed by summing all the points from items 1 to 9 of the PHQ-9M. Scores ranged from 0 to 27, with higher scores indicating greater severity of depression.^17^

#### Cox Regression With Time-Varying Covariates

We used Cox regression with time-varying covariates (accounting for multiple PHQ-9M screenings) to develop 3 models: (1) CR-9, a PHQ-9–based model using items 1 to 9; (2) CR-13, a PHQ-9M–based model using items 1 to 13; and (3) CR-3, a parsimonious model using 3 variables from PHQ-9M. Instead of choosing the 3 variables for the CR-3 model ad hoc, we followed a data-driven approach using the top 3 ranked multivariate HRs from 14 variables: 13 PHQ-9M items and PHQ-9 TS severity. A sensitivity analysis comparing different parsimonious models is described below. Both the CR-9 and CR-13 models included PHQ-9 TS severity as an additional variable.

For Cox regression modeling, we converted categorical to binary variables for items with values ranging from 0 to 3, that is, mapping an ordinal value (0 to 3) to 0 or 1, which minimizes the number of parameters and potential collinearity between variables. For PHQ-9 TS severity, we followed the common clinical practice, using 5 ordinal levels of PHQ-9 TS with a minor modification of the moderate depression threshold (from 10 to 11) adopted at CHOP, that is, minimal (0-4), mild (5-10), moderate (11-14), moderately severe (15-19), and severe (20-27) depression.

#### Modeling Process

We evaluated the predictive ability of the Cox regression models through the commonly used nested stratified 10-fold cross-validation.^20,21^ Each fold had stratified suicide visits at 4 prediction horizons: 30, 90, 180, and 365 days after the patients’ last PHQ-9M screening. To prevent data leakage, the Cox regression model built from a training fold predicted each patient’s risk for suicide attempts in a test fold using the linear predictor from a patient’s last PHQ-9M, that is, the product of Cox regression coefficients and PHQ-9M covariates. The model performance from the 10 test (holdout or blind) folds was reported. The PHQ-9 TS and the clinical rule were predetermined and did not require training. Similarly, the CR-3 model chose the top 3 variables from each training fold and evaluated in the test fold.

#### Missing Data Handling

We removed the patients who had only incomplete PHQ-9M screenings (336 [0.3%]) (Figure 1). Thus, the final cohort had patients with complete PHQ-9M data without missing values.

#### Evaluation Metrics and Prediction Time Horizons

Evaluation metrics included the area under the receiver operating characteristic curve (AUROC), the area under the precision recall curve (AUPRC), sensitivity (or recall), specificity, and positive predictive value (PPV or precision) at the 4 prediction horizons. We performed data bias and model fairness testing.^22^ For data bias, we measured data distributions in 2 demographic groups (sex and race and ethnicity). For model fairness, we measured prediction performance across the 2 demographic groups based on the best-performing model. Age-based analysis was not considered, since age in the non-SA group would be inconsistent to that of patients in the SA group due to the nature of multiple screenings and censoring. Note that the predictive models and screening strategies did not use any demographic variables.

eTable 2 in Supplement 1 lists commonly used prediction strategies. These included the STB rule and an alternative TS strategy (eg, using only the TS from the first 8 items [PHQ-8 TS]), in conjunction with item 9 or the combination of STB items (eg, PHQ-8 TS ≥10 and item 9 ≥1).

#### HRs and Correlation Analysis of PHQ-9M Items

To measure an item’s HR for the suicide attempt outcome based on endorsement of questionnaire items, we chose the majority answer within each item as the reference group. For example, for items with 4 categorical answers, the reference group was “not at all” (or score 0) (items 1-9), “not difficult at all” (item 11), or “no” (items 10, 12, and 13).

We measured unadjusted or univariate HRs (UHRs) and adjusted or multivariate HRs (AHRs) of the 13 PHQ-9M items based on the entire cohort without training-test splitting to assess the overall impact of screening items. Additionally, we reported the pairwise Spearman correlation between individual PHQ-9M items using the last PHQ-9M from each patient.

Following CR-3 modeling, we developed and evaluated 2 additional parsimonious models (CR-4 and CR-5) with the 4 and 5 top-ranked variables, respectively. All analyses were performed using the survival package of R, version 4.4.0 (R Program for Statistical Computing).^23^ Statistical significance was denoted as *P* < .05. All tests were 2 sided, calculated using the χ^2^ test for categorical variables, *t* test for numerical variables, and Delong test for models’ AUROC.

## Results

The final cohort included 272 402 PHQ-9M screenings from 130 028 patients (65 520 [50.4%] male, 64 498 female [49.6%], and 10 unknown [0.1%]) aged 12 to 17 years between 2016 and 2022. The ages of patients could not be obtained reliably due to multiple PHQ-9M screenings. Thus, a patient’s age would change depending on which screening was considered. Furthermore, due to censoring after the first observed suicide attempt, patients in the group without suicide attempts (non-SA group) were likely to have screenings at an older age compared with the SA group. In terms of race, 36 603 patients (28.2%) were Black; 70 776 (54.4%) were White; and 21 256 (16.3%) were categorized as other race (including American Indian or Alaska Native, Asian, Indian from the subcontinent, and Native Hawaiian or Other Pacific Islander). A total of 10 015 patients (7.7%) were Hispanic or Latino. A total of 549 (0.4%) had a first suicide attempt within 1 year from their last screening (SA group) (Figure 1). The initial cohort had 130 788 patients with 274 893 PHQ-9M screenings during outpatient encounters; 80 340 (61.8%) of the 130 028 patients were screened more than once (mean [SD] of 2.1 [1.1] screenings per person). The median time to the closest suicide attempt from PHQ-9M screening was 172 (IQR, 82-267) days. eFigures 1 and 2 in Supplement 1 show boxplots with days to suicide attempt across 8 subgroups and cumulated counts of suicide attempts after the last screening.

Table 1 summarizes demographic characteristics with odds ratios (ORs) of the final cohort. In comparing the SA with the non-SA groups, female adolescents were at a higher risk of suicide attempts than male adolescents (OR, 3.81 [3.12-4.70]; *P* < .001), and Black patients were at higher risk compared with White patients (OR, 1.48 [95% CI, 1.23-1.78]; *P* < .001). eFigures 3 to 5 in Supplement 1 show PHQ-9 TS severity across races and values of items 13 and 9. eTable 1 in Supplement 1 compares patients with a single screening (49 686 patients) and those with multiple screenings (80 340 patients with 222 714 screenings) in PHQ-9 TS and the 3 STB items during the study period. Those with a single screening had slightly higher levels of mean (SD) PHQ-9 TS (3.38 [4.29] vs 3.00 [4.03]; *P* < .001; difference of means, 0.38 [95% CI, 0.34-0.43]), and more frequent positive answers to items 9 (5.7% vs 4.5%; *P* < .001) and 12 (3.3% vs 2.4%; *P* < .001). The *P* values were measured by the χ^2^ test with 1 *df* between categorical variables and the unpaired, 2-sided *t* test between continuous variables.

**Table 1.** Patient-Based Demographic Characteristics of Patients Who Completed PHQ-9M Questionnaires

Variable	Patient group^a^		Odds ratio (95% CI)
	Non-SA (n = 129 479)	SA (n = 549)	
Sex			
Female	64 065 (49.5)	433 (78.9)	3.81 (3.12-4.7)
Male	65 404 (50.5)	116 (21.1)	1 [Reference]
Unknown	10 (0.01)	0	NA
Race			
Black	36 403 (28.1)	200 (36.4)	1.48 (1.23-1.78)
White	70 515 (54.5)	261 (47.5)	1 [Reference]
Other^b^	21 174 (16.4)	82 (14.9)	1.05 (0.81-1.33)
Unknown	1387 (1.1)	6 (1.1)	1.17 (0.46-2.4)
Hispanic or Latino ethnicity			
Yes	9962 (7.7)	53 (9.7)	1.29 (0.96-1.69)
No	118 701 (91.7)	491 (89.4)	1 [Reference]
Unknown	816 (0.6)	5 (0.9)	1.48 (0.53-3.21)
Age, y^c^			
12-15	NA	400 (72.9)	NA
16-17	NA	149 (27.1)	NA
Age at PHQ-9M screening, median (IQR)^c^	NA	14 (13-16)	NA

Abbreviations: NA, not applicable; PHQ-9M, Patient Health Questionnaire–9 modified for teens; SA, suicide attempt.

^a^ Patients were grouped in the SA or non-SA group based on SAs within 1 year following PHQ-9M administration. Unless otherwise indicated, data are expressed as No. (%) of patients.

^b^ Includes American Indian or Alaska Native, Asian, Indian, and Native Hawaiian or Other Pacific Islander.

^c^ The age of patients in the non-SA group could not be obtained reliably due to multiple PHQ-9M screenings.

### Prediction Performance of PHQ-9M Items

eTable 2 in Supplement 1 summarizes performance of commonly used models to predict suicide attempts (eg, STB, PHQ-8 TS with STB items), the PHQ-9 TS using different cutoff points, and the contribution of individual PHQ-9M items; item 9 with a score of 3 (nearly every day) had the highest PPV of 4.8% (95% CI, 3.2%-6.6%) and specificity of 99.6% (95% CI, 99.5%-99.6%) across all 13 items and screening strategies using PHQ-9 or PHQ-8 TS and/or the STB rule. eFigure 4 in Supplement 1 shows the distributions of item 13 responses in the final cohort. eTables 3 to 5 in Supplement 1 further evaluated prediction performance of 3 STB items across the 4 prediction horizons. Decreasing sensitivity and increasing PPV were observed when the length of a prediction horizon increased. Item 9 had a relatively lower missing report rate; 67.9% of adolescents with suicide attempts within 1 year did not report a positive value, compared with 78.0% for item 12 and 71.2% for item 13.

### HRs and Correlation Among PHQ-9M Items and PHQ-9 TS

**Figure 2.**
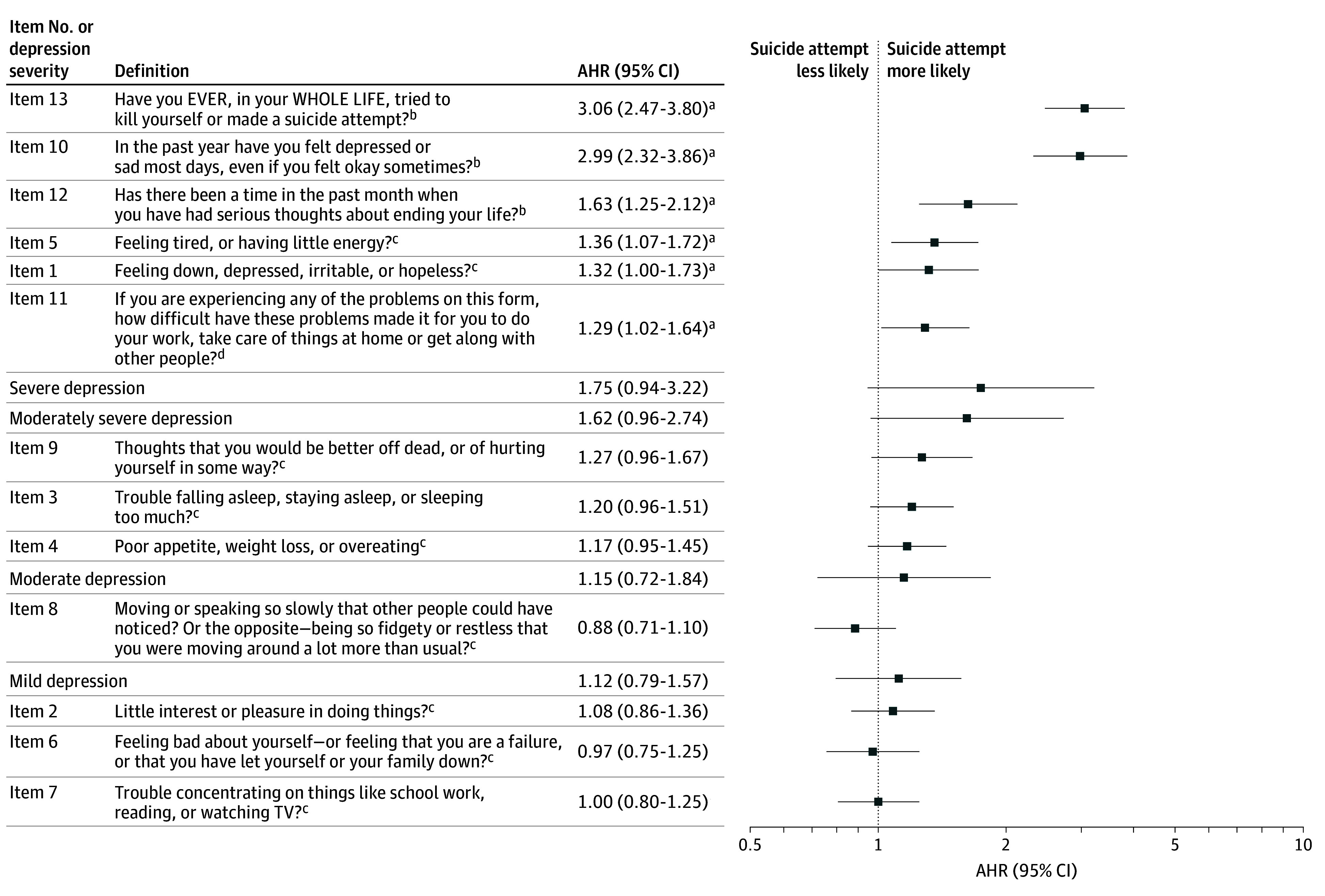
Ranked Adjusted Hazard Ratios (AHRs) for All 13 Items in the Patient Health Questionnaire–9 Modified for Teens (PHQ-9M) and the Severity of the Patient Health Questionnaire–9 Total Score (PHQ-9 TS) The hazard ratios for items 1 to 13 have a reference group of score 0 or "no." The hazard ratios for the PHQ-9 total score severity have minimal (PHQ-9 TS <5) as a reference group. Mild indicates a PHQ-9 TS between 5 and 10; moderate, a PHQ-9 TS between 11 and 14; moderately severe, a PHQ-9 TS between 15 and 19; and severe, a PHQ-9 TS between 20 and 27. ^a^Significant hazard ratio (*P* < .05) measured via the Wald test of Cox proportional hazards regression coefficients. ^b^Yes or no. ^c^Scoring: 0, not at all; 1, several days; 2, more than half the days; or 3, nearly every day. ^d^Scored as not difficult at all, somewhat difficult, very difficult, or extremely difficult.

We measured AHRs of 13 PHQ-9M items and 5 PHQ-9 TS severity levels (mild, moderate, moderately severe, and severe, with minimal as the reference) as shown in Figure 2. Items 13, 10, and 12 were ranked as the top 3 variables associated with future suicide attempts, with AHRs of 3.06 (95% CI 2.47-3.80) for item 13, 2.99 (95% CI, 2.32-3.86) for item 10, and 1.63 (95% CI, 1.25-2.12) for item 12. eFigure 6 in Supplement 1 shows the paired Spearman correlation matrix among the 13 items. eFigure 7 in Supplement 1 shows UHRs; item 9 had a higher UHR but a substantially lower AHR (Figure 2) (9.77 [95% CI, 8.16-11.69] vs 1.27 [95% CI, 0.96-1.67]), possibly explained by a moderate correlation with item 12 (0.46).

### Predictive Model Performance

**Figure 3.**
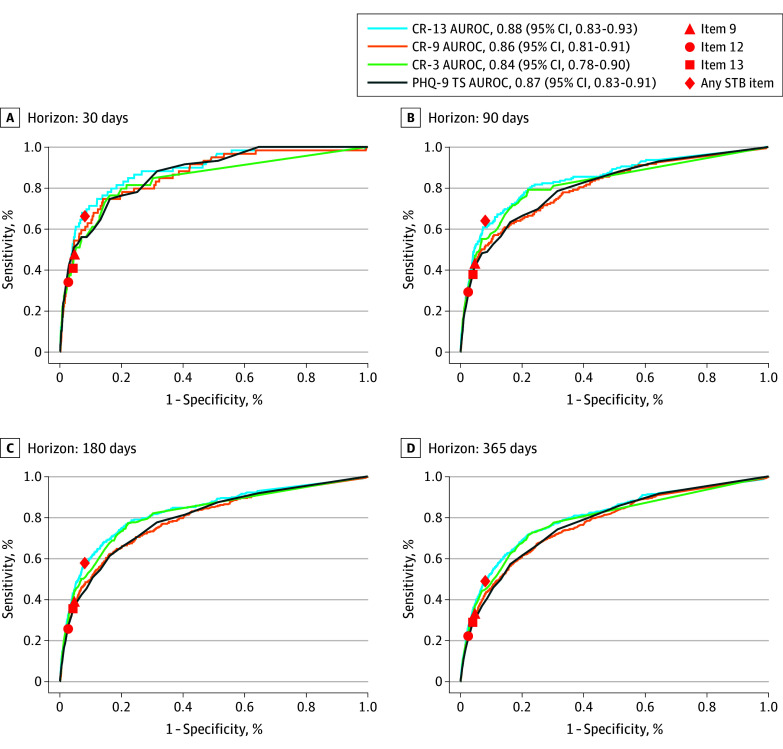
The Area Under the Receiver Operating Characteristics Curves (AUROC) of 5 Predictors Derived from the Patient Health Questionnaire–9 Modified for Teens (PHQ-9M) for the Prediction of Subsequent Suicide Attempts Four prediction horizons following PHQ-9M questionnaires were evaluated. The group without suicide attempts is the same across horizons (n = 129 479). The size of the group with suicide attempts varies across horizons as follows: 59 patients at 30 days, 158 at 90 days, 294 at 180 days, and 549 at 365 days. PHQ-9 item 9 measures the frequency of current suicidal ideation or thoughts of self-harm; PHQ-9M item 12, serious suicidal ideation in the past month; and PHQ-9M item 13, lifetime history of suicide attempts. Any suicidal thought or behavior (STB) item is defined as endorsement of any of the 3 STB items (item 9, ≥1; item 12, yes; or item 13, yes). CR-3 indicates Cox proportional hazards regression model built from top 3 of 14 variables including 13 PHQ-9M items and PHQ-9 total score (TS) severity; CR-9, Cox proportional hazards regression model built from items 1 to 9 on the PHQ-9M (same 9 items on PHQ-9) and PHQ-9 TS severity; and CR-13, Cox proportional hazards regression model built from all 13 items on PHQ-9M and PHQ-9 TS severity.

Table 2 summarizes model performance (AUROC and AUPRC) among the 3 regression models and the PHQ-9 TS for predicting suicide attempts. The CR-13 model performed best (AUROC, 0.80 [95% CI, 0.78-0.82]) and outperformed the PHQ-9 TS (AUROC, 0.77 [95% CI, 0.75-0.79]; *P* < .001) and the CR-9 model (AUROC, 0.77 [95% CI, 0.75-0.79]; *P* < .001) in the 365-day prediction horizon. The AUPRC in the CR-9 model was 0.02 (95% CI, 0.02-0.03); in the CR-13 model, 0.03 (95% CI, 0.02-0.03); and in the CR-3 model, 0.02 (95% CI, 0.02-0.03). Figure 3 shows AUROC plots of the 3 regression models, PHQ-9 TS, the STB rule, and 3 STB items across 4 prediction horizons (30, 90, 180, and 365 days from the last PHQ-9M screening).

**Table 2.** Predictive Performance Evaluation Among 3 Cox Proportional Hazards Regression Models and the PHQ-9 TS for Suicide Attempts in 4 Prediction Horizons Following PHQ-9M Screening

Prediction horizon	PHQ-9 TS	CR-13	CR-9	CR-3^a^
30 d				
AUROC (95% CI)	0.87 (0.83-0.91)	0.88 (0.83-0.93)^b^	0.86 (0.81-0.91)	0.84 (0.78-0.90)^c^
AUPRC (95% CI)	0.01 (0.00-0.01)	0.01 (0.00-0.02)	0.00 (0.00-0.01)	0.01 (0.00-0.01)
90 d				
AUROC (95% CI)	0.81 (0.77-0.84)^c^	0.84 (0.81-0.88)^b^	0.80 (0.76-0.84)^c^	0.82 (0.78-0.86)^c^
AUPRC (95% CI)	0.01 (0.01-0.01)	0.01 (0.01-0.02)	0.01 (0.01-0.01)	0.01 (0.01-0.02)
180 d				
AUROC (95% CI)	0.79 (0.77-0.82)^c^	0.83 (0.80-0.86)^b^	0.79 (0.76-0.82)^c^	0.82 (0.79-0.84)^c^
AUPRC (95% CI)	0.01 (0.01-0.02)	0.02 (0.02-0.03)	0.01 (0.01-0.02)	0.02 (0.01-0.02)
365 d				
AUROC (95% CI)	0.77 (0.75-0.79)^c^	0.80 (0.78-0.82)^b^	0.77 (0.75-0.79)^c^	0.79 (0.76-0.81)^c^
AUPRC (95% CI)	0.02 (0.02-0.02)	0.03 (0.02-0.03)	0.02 (0.02-0.03)	0.02 (0.02-0.03)

Abbreviations: AUROC, area under the receiver operating characteristics curve; AUPRC, area under the precision recall curve; CR-3, Cox proportional hazards regression model built from top 3 variables from 14 variables including 13 Patient Health Questionnaire–9 modified for teens (PHQ-9M) items and Patient Health Questionnaire–9 total score (PHQ-9 TS) severity; CR-9, Cox proportional hazards regression model built from items 1 to 9 on the PHQ-9M (same 9 items on PHQ-9) and PHQ-9 TS severity; CR-13, Cox proportional hazards regression model built from all 13 items on PHQ-9M and PHQ-9 TS severity (reference model).

^a^ The top variables were selected from the training data fold.

^b^ Indicates the best performance across models within a prediction horizon.

^c^ Statistically significant difference (Delong test *P* < .05) compared with model CR-13. eTable 7 in Supplement 1 provides the 95% CI of the AUROC and AUPRC difference between models.

### Data Bias and Model Fairness

Of the 549 patients in the SA group, 433 (78.9%) were female. Black adolescents (n = 36 603) had the highest suicide attempt prevalence, 0.55% (n = 200), across races with statistical significance compared with White adolescents (suicide attempt prevalence: 0.37%) (Table 1).

The CR-13 model showed good fairness across race and sex subgroups. It had no statistically significant bias across 2 main race subgroups with AUROCs of 0.79 (95% CI, 0.76-0.83) for Black and 0.82 (95% CI, 0.79-0.85) for White adolescents (*P* = .29; AUROC difference, −0.03 [95% CI, −0.06 to 0.02]). It also performed similarly between both sex subgroups with AUROCs of 0.79 (95% CI, 0.76-0.81) for female and 0.75 (95% CI, 0.70-0.80) for male adolescents (*P* = .21; AUROC difference, 0.04 [95% CI, −0.10 to 0.02]).

### Sensitivity Analysis on Parsimonious Models

eTable 6 in Supplement 1 lists prediction performance of the 3 parsimonious models (CR-3, CR-4, and CR-5). The AUROC for the parsimonious CR-3 model was 0.79 (95% CI, 0.76-0.81), and performed similarly to the parsimonious models CR-4 and CR-5 (with the same AUROC of 0.79 [95% CI, 0.77-0.81]) without a statistically significant difference.

## Discussion

In this retrospective single-center cohort study including 130 028 adolescents (272 529 screenings), we compared clinical utility between PHQ-9M and PHQ-9 (consisting of the first 9 core items of PHQ-9M) to identify patients at risk of subsequent suicide attempts within 365 days following the PHQ-9M screening. We developed Cox proportional hazards modeling of multiple screenings for a patient during the study period. The regression model, CR-13 (AUROC, 0.80 [95% CI, 0.78-0.82]), using all 13 items of PHQ-9M, outperformed the CR-9 model (AUROC, 0.77 [95% CI, 0.75-0.79]), using 9 PHQ-9 items, with statistical significance across 3 prediction horizons (90, 180, and 365 days following PHQ-9M). We further developed data-driven parsimonious models for potential clinical use aimed at having 3 to 5 variables and reaching a similar performance to that of the full model (CR-13). The parsimonious CR-3 model (AUROC, 0.79 [95% CI, 0.76-0.81]), using 3 top-ranked variables based on AHRs from training folds, had a similar performance to the other 2 parsimonious models (CR-4 and CR-5) (with the same AUROC of 0.79 [95% CI, 0.77-0.81]) and CR-13. The overall 3 top-ranked variables were the supplemental items 13, 10, and 12, respectively (Figure 2). Moreover, all 4 supplemental items (10-13) had statistically significant AHRs. Thus, the clinical utility of the PHQ-9M for suicide risk prediction was above and beyond information gleaned from PHQ-9, which highlights the importance of incorporating the supplemental items into clinical risk determinations and decision-making. To our knowledge, this is the first study comparing clinical utility between PHQ-9M and PHQ-9 for subsequent prediction of adolescent suicide attempts.

This study validates the significance of historical suicidal behavior (item 13), depression (item 10), and recent suicidal ideation (item 12) reflected in the literature.^9,22,24,25,26^ Item 13 was the strongest risk factor, consistent with the literature, followed by items 10 and 12.^24,25,26^

The parsimonious model CR-3 has advantages. First, it used only 3 variables with a slightly lower AUROC compared with CR-13 (0.79 vs 0.80) although the difference had statistical significance due to the large cohort size. Second, unlike the STB rule (triggered by any positive STB items) or individual STB items with a specific sensitivity or specificity (Figure 3), the CR-3 model is flexible in choosing different sensitivity or specificity values. Thus, the CR-3 model could serve as an alternative to the STB rule. In the sensitivity analysis, the CR-3 model performed similarly to the CR-4 and CR-5 models without statistical significance (eTable 6 in Supplement 1).

All the models demonstrated a decreasing AUROC when the prediction horizon was increased. This could be attributed to the challenge of a long-term prediction, given that risk of suicide attempts could change over time due to clinical interventions or social determinants of health (eg, family support).

Item 9 had a much lower AHR (1.27 [95% CI, 0.96-1.67]) vs UHR (9.77 [95% CI, 8.16-11.69]). Such a large difference may be explained by the moderate correlation (0.46) with item 12 (eFigure 6 in Supplement 1) and its less specific nature (including suicidal thoughts and behaviors as well as nonsuicidal self-injury) compared with items 12 and 13. Item 9 had a relatively lower missing report rate; 67.9% of adolescents with suicide within 1 year did not report a positive value, compared with 78.0% for item 12 and 71.2% for item 13. These findings suggest that adding these items to the PHQ-9 can help to identify youths at risk for suicide attempts who, despite a negative screening result on item 9, attempt suicide within 30 days of screening.^27^

Our study found that female adolescents were at a higher risk of suicide attempts than male adolescents (odds ratio, 3.81 [3.12-4.70]). This finding is similar to those of recent studies in high school students and adolescents.^16,28^

Accurate prediction of subsequent suicide attempts within 1 year remains extremely challenging, given the very low prevalence of documented suicide attempts in EHRs (0.4%). Although the AUPRC^29^ of the PHQ-9M–based models (CR-3, CR-9, and CR-13) was low, it was about 5 to 7 times higher than the prevalence of suicide attempts. However, future studies are required to further improve the PPV in conjunction with the net benefit after the relative costs of interventions for clinical use are considered.^30^ Recent developments in using machine learning models and natural language processing of EHR data could potentially serve as an additional approach for risk screening in conjunction with PHQ-9M.^22^

### Limitations

This study has limitations. Patients were included from a single regional health care system in the northeastern US, and thus findings may not generalize to other health care settings. Additionally, patients may have completed the PHQ-9M or visits for suicide attempts in other health care systems that were not captured in our system. Patients aged 12 to 15 and 17 years were not universally screened with the PHQ-9M questionnaire until November 2017. We did not try specific strategies for improving prediction performance, such as up or down sampling between screenings with and without suicide attempts, or penalty weighting on missed prediction of suicide attempts, given the focus on model comparison.

## Conclusions

In this cohort study, PHQ-9M screening with supplemental items enabled improved performance in predicting subsequent suicide attempts compared with the 9 core items of PHQ-9M (ie, the PHQ-9). The historical suicidal behavior (item 13) was the strongest risk factor among 13 items on the PHQ-9M. A parsimonious model using the top 3 risk factors, suicidal behavior (item 13), depression (item 10), and suicidal thoughts (item 12), may serve as an alternative to the conventional STB rule (items 9, 12, or 13). With the challenge of achieving low prevalence of suicide attempts, further development of suicide risk prediction that uses EHRs and PHQ-9M screening results may further improve prediction performance.

## Notes

Supplement 1. **eMethods.** PHQ-9M Measures **eTable 1.** PHQ-9 Total Score (TS) and Levels of Suicidal Thoughts and Behaviors (Items 9, 12, and 13) in All Patients and Patients With 1 vs Multiple Screenings During the Study Period **eTable 2.** PHQ-9M and Ad Hoc Rule Screening Performance for Predicting Subsequent Suicide Attempts Within 1 Year **eTable 3.** Prediction Performance of PHQ-9 Item 9 at 4 Prediction Horizons Following the PHQ-9M Screening **eTable 4.** Prediction Performance of PHQ-9M Item 12 at 4 Prediction Horizons Following the PHQ-9M Screening **eTable 5.** Prediction Performance of PHQ-9M Item 13 at 4 Prediction Horizons Following the PHQ-9M Screening **eTable 6.** Sensitivity Analysis of 3 Parsimonious Models in 4 Prediction Horizons Following PHQ-9M Screening **eTable 7.** Confidence Intervals of Performance Differences Between Predictive Models in 4 Prediction Horizons Following PHQ-9M Screening **eFigure 1.** Boxplots of Days to the First (Closest) Suicide Attempt After the PHQ-9M Screening in Different Subgroups Based on Race, Sex, and Age **eFigure 2.** A Cumulative Plot With Counts of Suicide Attempts (n = 549) by Days After PHQ-9M Screening **eFigure 3.** Distribution of Races Across 5 Depression Severity Groups Based on PHQ-9 Total Scores **eFigure 4.** Distributions of PHQ-9 Total Score Severity Groups in the Final Cohort and Screenings With Positive Item 13 (Self-Reported Suicide Attempt History) **eFigure 5.** Distribution of Item 9 Scores (Self-Reported Suicide Ideation) Across 5 PHQ-9 Severity Groups **eFigure 6.** Pairwise Spearman Correlation Matrix of Individual PHQ-9M Items **eFigure 7.** Ranked Unadjusted Hazard Ratios (UHRs) for All 13 Items in the PHQ-9M Questionnaire and the Severity of the PHQ-9 Total Score **eFigure 8.** Precision Recall Curves of 5 Predictors Derived From the PHQ-9M Questionnaire for the Prediction of Subsequent Suicide Attempts
eReferences.

Supplement 2.
Data Sharing Statement
