# Supplementary material for: Risk for Suicide Attempts Assessed Using the Patient Health Questionnaire–9 Modified for Teens
Source: JAMA Netw Open. 2024 Oct 8;7(10):e2438144. doi: 10.1001/jamanetworkopen.2024.38144 (PMC11581555; doi:10.1001/jamanetworkopen.2024.38144)
Supplement: Supplement 1. — eMethods. PHQ-9M Measures eTable 1. PHQ-9 Total Score (TS) and Levels of Suicidal Thoughts and Behaviors (Items 9, 12, and 13) in All Patients and Patients With 1 vs Multiple Screenings During the Study Period eTable 2. PHQ-9M and Ad Hoc Rule Screening Performance for Predicting Subsequent Suicide Attempts Within 1 Year eTable 3. Prediction Performance of PHQ-9 Item 9 at 4 Prediction Horizons Following the PHQ-9M Screening eTable 4. Prediction Performance of PHQ-9M Item 12 at 4 Prediction Horizons Following the PHQ-9M Screening eTable 5. Prediction Performance of PHQ-9M Item 13 at 4 Prediction Horizons Following the PHQ-9M Screening eTable 6. Sensitivity Analysis of 3 Parsimonious Models in 4 Prediction Horizons Following PHQ-9M Screening eTable 7. Confidence Intervals of Performance Differences Between Predictive Models in 4 Prediction Horizons Following PHQ-9M Screening eFigure 1. Boxplots of Days to the First (Closest) Suicide Attempt After the PHQ-9M Screening in Different Subgroups Based on Race, Sex, and Age eFigure 2. A Cumulative Plot With Counts of Suicide Attempts (n = 549) by Days After PHQ-9M Screening eFigure 3. Distribution of Races Across 5 Depression Severity Groups Based on PHQ-9 Total Scores eFigure 4. Distributions of PHQ-9 Total Score Severity Groups in the Final Cohort and Screenings With Positive Item 13 (Self-Reported Suicide Attempt History) eFigure 5. Distribution of Item 9 Scores (Self-Reported Suicide Ideation) Across 5 PHQ-9 Severity Groups eFigure 6. Pairwise Spearman Correlation Matrix of Individual PHQ-9M Items eFigure 7. Ranked Unadjusted Hazard Ratios (UHRs) for All 13 Items in the PHQ-9M Questionnaire and the Severity of the PHQ-9 Total Score eFigure 8. Precision Recall Curves of 5 Predictors Derived From the PHQ-9M Questionnaire for the Prediction of Subsequent Suicide Attempts eReferences. [file jamanetwopen-e2438144-s001.pdf]

Supplementary Online Content

Tsui F, Ruiz VM, Ryan ND, et al. Risk for suicide attempts assessed using the Patient Health Questionnaire–9 modified for teens. *JAMA Netw Open*. 2024;7(10):e2438144. doi:10.1001/jamanetworkopen.2024.38144

eMethods. PHQ-9M Measures
eTable 1. PHQ-9 Total Score (TS) and Levels of Suicidal Thoughts and Behaviors (Items 9, 12, and 13) in All Patients and Patients With 1 vs Multiple Screenings During the Study Period
eTable 2. PHQ-9M and Ad Hoc Rule Screening Performance for Predicting Subsequent Suicide Attempts Within 1 Year
eTable 3. Prediction Performance of PHQ-9 Item 9 at 4 Prediction Horizons Following the PHQ-9M Screening
eTable 4. Prediction Performance of PHQ-9M Item 12 at 4 Prediction Horizons Following the PHQ-9M Screening
eTable 5. Prediction Performance of PHQ-9M Item 13 at 4 Prediction Horizons Following the PHQ-9M Screening
eTable 6. Sensitivity Analysis of 3 Parsimonious Models in 4 Prediction Horizons Following PHQ-9M Screening
eTable 7. Confidence Intervals of Performance Differences Between Predictive Models in 4 Prediction Horizons Following PHQ-9M Screening
eFigure 1. Boxplots of Days to the First (Closest) Suicide Attempt After the PHQ-9M Screening in Different Subgroups Based on Race, Sex, and Age
eFigure 2. A Cumulative Plot With Counts of Suicide Attempts (n = 549) by Days After PHQ-9M Screening

eFigure 3. Distribution of Races Across 5 Depression Severity Groups Based on PHQ-9 Total Scores

eFigure 4. Distributions of PHQ-9 Total Score Severity Groups in the Final Cohort and Screenings With Positive Item 13 (Self-Reported Suicide Attempt History)

eFigure 5. Distribution of Item 9 Scores (Self-Reported Suicide Ideation) Across 5 PHQ-9 Severity Groups

eFigure 6. Pairwise Spearman Correlation Matrix of Individual PHQ-9M Items
eFigure 7. Ranked Unadjusted Hazard Ratios (uHRs) for All 13 Items in the PHQ-9M Questionnaire and the Severity of the PHQ-9 Total Score
eFigure 8. Precision Recall Curves of 5 Predictors Derived From the PHQ-9M Questionnaire for the Prediction of Subsequent Suicide Attempts
eReferences.

This supplementary material has been provided by the authors to give readers additional information about their work.

© 2024 Tsui F et al. *JAMA Network Open*.

© 2024 Tsui F et al. *JAMA Network Open*.

eMethods. PHQ-9M Measures

The Patient Health Questionnaire Modified for Teens (PHQ-9M) is an established self-report depression screening commonly used in primary care settings during annual wellness visits for adolescents. During the visit, before seeing the clinician, each adolescent was asked to complete the self-reporting PHQ-9M through an electronic tablet, and the information was automatically uploaded to the EHR system. The PHQ-9M retains the nine core items (items 1-9) from the common depression screening instrument PHQ-9, with slight modifications to enhance relevance to youth depression, and 4 supplemental items (items 10-13). The slight modifications include the ordering and minor wording changes of some items (e.g., item 2 "Feeling down, depressed, or hopeless" on PHQ-9 becomes item 1 "Feeling down, depressed, irritable, or hopeless" on PHQ-9M). The first 9 items match the Diagnostic and Statistical Manual of Mental Disorder Fourth Edition (DSM-IV)[1] criteria of major depression; each item has a 4-point score with 0 (not at all), 1 (several days), 2 (more than half the days), and 3 (nearly every day). Individual scores from items 1 to 9 are summed up to calculate the PHQ-9 total score (PHQ-9 TS) ranging from 0 to 27.[2.] The PHQ-9 TS has five categorical depression severity: minimal depression (0-4), mild depression (5-10), moderate depression (11-14), Moderately severe depression (15-19), and severe depression (20-27).[3] The four supplemental items address depression severity/impairment (items 10-11) and assess suicide risk (items 12-13).[4] The following list shows the full 13 items of PHQ-9M.

Item 1. Feeling down, depressed, irritable, or hopeless? 0: not at all; 1: several days; 2: more than half the days; or 3: nearly every day

Item 2. Little interest or pleasure in doing things? 0: not at all; 1: several days; 2: more than half the days; or 3: nearly every day

Item 3. Trouble falling asleep, staying asleep, or sleeping too much? 0: not at all; 1: several days; 2: more than half the days; or 3: nearly every day

Item 4. Poor appetite, weight loss, or overeating? 0: not at all; 1: several days; 2: more than half the days; or 3: nearly every day

Item 5. Feeling tired, or having little energy? 0: not at all; 1: several days; 2: more than half the days; or 3: nearly every day

Item 6. Feeling bad about yourself — or feeling that you are a failure, or that you have let yourself or your family down? 0: not at all; 1: several days; 2: more than half the days; or 3: nearly every day

Item 7. Trouble concentrating on things like school work, reading, or watching TV? 0: not at all; 1: several days; 2: more than half the days; or 3: nearly every day

Item 8. Moving or speaking so slowly that other people could have noticed? Or the opposite — being so fidgety or restless that you were moving around a lot more than usual? 0: not at all; 1: several days; 2: more than half the days; or 3: nearly every day

Item 9. Thoughts that you would be better off dead, or of hurting yourself in some way? 0: not at all; 1: several days; 2: more than half the days; or 3: nearly every day

Item 10. (supplemental item) In the past year have you felt depressed or sad most days, even if you felt okay sometimes? Yes or no

Item 11. (supplemental item) If you are experiencing any of the problems on this form, how difficult have these problems made it for you to do your work, take care of things at home or get along with other people? (supplemental item)

Item 12. Has there been a time in the past month when you have had serious thoughts about ending your life? Yes or no

Item 13. (supplemental item) Have you EVER, in your WHOLE LIFE, tried to kill yourself or made a suicide attempt? Yes or no

© 2024 Tsui F et al. *JAMA Network Open*.

eTable 1. PHQ-9 Total Score (TS) and Levels of Suicidal Thoughts and Behaviors (Items 9, 12, and 13) in All Patients and Patients With 1 vs Multiple Screenings During the Study Period

Item	All Patients (n=80,340 patients; 272,402 screenings)	Patients with one screening (n=49,688 screenings)	Patients with multiple screenings (n=80,340 patients; 222,714 screenings)	P-value (one vs. multiple screenings)
PHQ-9 TS average (std)	3.07 (4.08)	3.38 (4.29)	3.00 (4.03)	<0.001
Item 9 ≥1 (%)	12,914 (4.74)	2,836 (5.71)	10,078 (4.53)	<0.01
Item 12 = Yes (%)	6,978 (2.56)	1,630 (3.28)	5,348 (2.40)	<0.01
Item 13 = Yes (%)	9,234 (3.39)	2,015 (4.06)	7,219 (3.24)	<0.01

PHQ-9: Patient health questionnaire; Item 12: Has there been a time in the past month when you have had serious thoughts about ending your life?; Item 13: Have you EVER, in your WHOLE LIFE, tried to kill yourself or made a suicide attempt?; Items 12 and 13 are additional questionnaire items added to the Patient health questionnaire modified for teens (PHQ-9M)
The p values were estimated by the Chi-square test for categorical variables and the T-test for the continuous variable.

eTable 2. PHQ-9M and Ad Hoc Rule Screening Performance for Predicting Subsequent Suicide Attempts Within 1 Year

Question	Value	Sensitivity (%)	Specificity (%)	PPV (%)	F1* (%)
Item 1: Feeling down, depressed, irritable, or hopeless?	≥ 1	62.7 (58.7-66.5)	78.2 (77.9-78.4)	1.2 (1.1-1.3)	2.4 (2.2-2.5)
	≥ 2	31.3 (27.7-35.3)	94.3 (94.2-94.5)	2.3 (2.0-2.6)	4.3 (3.8-4.8)
	= 3	16.6 (13.5-19.9)	98.1 (98.0-98.1)	3.5 (2.8-4.1)	5.8 (4.7-6.8)
Item 2: Little interest or pleasure in doing things?	≥ 1	60.8 (56.8-64.8)	72.3 (72.0-72.5)	0.9 (0.9-1.0)	1.8 (1.7-1.9)
	≥ 2	27.9 (24.0-31.7)	90.6 (90.4-90.7)	1.2 (1.1-1.4)	2.4 (2.1-2.7)
	= 3	12.9 (10.2-15.8)	96.0 (95.9-96.1)	1.3 (1.1-1.6)	2.4 (1.9-3.0)
Item 3: Trouble falling asleep, staying asleep, or sleeping too much?	≥ 1	68.3 (64.5-71.9)	62.9 (62.6-63.1)	0.8 (0.7-0.8)	1.5 (1.4-1.6)
	≥ 2	46.3 (42.1-50.5)	85.2 (85.0-85.4)	1.3 (1.2-1.4)	2.5 (2.3-2.8)
	= 3	25.1 (21.5-28.8)	93.0 (92.8-93.1)	1.5 (1.3-1.7)	2.8 (2.4-3.2)
Item 4: Poor appetite, weight loss, or overeating?	≥ 1	48.5 (44.3-52.6)	80.4 (80.1-80.6)	1.0 (0.9-1.1)	2.0 (1.9-2.2)
	≥ 2	26.6 (22.9-30.2)	92.9 (92.8-93.0)	1.6 (1.3-1.8)	3.0 (2.5-3.4)
	= 3	10.6 (8.0-13.1)	97.2 (97.1-97.3)	1.6 (1.2-2.0)	2.8 (2.1-3.4)
Item 5: Feeling tired, or having little energy?	≥ 1	**72.7 (69.2-76.1)**	59.5 (59.3-59.8)	0.8 (0.7-0.8)	1.5 (1.4-1.6)
	≥ 2	39.3 (35.5-43.4)	88.0 (87.8-88.1)	1.4 (1.2-1.5)	2.6 (2.4-2.9)
	= 3	20.8 (17.5-24.2)	95.2 (95.0-95.3)	1.8 (1.5-2.1)	3.3 (2.8-3.8)

© 2024 Tsui F et al. *JAMA Network Open*.

Item 6: Feeling bad about yourself — or feeling that you are a failure, or that you have let yourself or your family down?	≥ 1	53.7 (49.7-57.7)	82.6 (82.4-82.8)	1.3 (1.2-1.4)	2.5 (2.3-2.7)
	≥ 2	29.5 (25.7-33.3)	94.2 (94.0-94.3)	2.1 (1.8-2.4)	3.9 (3.4-4.4)
	= 3	14.2 (11.5-17.3)	97.8 (97.7-97.8)	2.6 (2.1-3.2)	4.4 (3.6-5.4)
Item 7: Trouble concentrating on things like school work, reading, or watching TV?	≥ 1	59.6 (55.6-63.6)	69.2 (68.9-69.4)	0.8 (0.8-0.9)	1.6 (1.5-1.7)
	≥ 2	34.8 (31.0-38.8)	88.4 (88.2-88.5)	1.3 (1.1-1.4)	2.4 (2.1-2.7)
	= 3	18.2 (14.9-21.5)	94.7 (94.5-94.8)	1.4 (1.2-1.7)	2.7 (2.2-3.1)
Item 8: Moving or speaking so slowly that other people could have noticed? Or the opposite — being so fidgety or restless that you were moving around a lot more than usual?	≥ 1	32.8 (29.1-36.8)	88.1 (87.9-88.3)	1.2 (1.0-1.3)	2.2 (2.0-2.5)
	≥ 2	15.8 (12.9-19.1)	95.9 (95.8-96.0)	1.6 (1.3-1.9)	2.9 (2.4-3.5)
	= 3	6.6 (4.6-8.6)	98.4 (98.3-98.5)	1.7 (1.2-2.2)	2.7 (1.9-3.5)
Item 9: Thoughts that you would be better off dead, or of hurting yourself in some way?	≥ 1	32.1 (28.2-35.7)	95.3 (95.2-95.4)	2.8 (2.5-3.1)	5.2 (4.5-5.7)
	≥ 2	12.2 (9.5-15.1)	98.7 (98.7-98.8)	3.9 (3.1-4.9)	5.9 (4.6-7.3)
	= 3	5.1 (3.5-7.1)	**99.6 (99.5-99.6)**	**4.8 (3.2-6.6)**	4.9 (3.3-6.8)
Item 10: In the past year have you felt depressed or sad most days, even if you felt okay sometimes?	YES	67.8 (63.9-71.4)	79.5 (79.2-79.7)	1.4 (1.3-1.5)	2.7 (2.5-2.8)
Item 11: If you are experiencing any of the problems on this form, how difficult have these problems made it for you to do your work, take care of things at home or get along with other people?	≥ 1	61.6 (57.4-65.4)	76.8 (76.6-77.1)	1.1 (1.0-1.2)	2.2 (2.0-2.3)
	≥ 2	22.0 (18.6-25.3)	96.4 (96.3-96.5)	2.5 (2.2-2.9)	4.6 (3.9-5.2)
	= 3	7.3 (5.3-9.5)	99.3 (99.2-99.3)	4.1 (2.9-5.4)	5.3 (3.7-6.9)
Item 12: Has there been a time in the past month when you have had serious thoughts about ending your life?	YES	22.0 (18.8-25.5)	97.3 (97.2-97.4)	3.4 (2.9-3.9)	5.8 (5.0-6.8)
Item 13: Have you EVER, in your WHOLE LIFE, tried to kill yourself or made a suicide attempt?	YES	28.8 (25.1-32.6)	96.0 (95.9-96.1)	2.9 (2.6-3.3)	5.3 (4.7-6.0)
PHQ-9 total score ≥ 5 (Mild to Severe)	TRUE	66.7 (62.8-70.3)	74.9 (74.7-75.2)	1.1 (1.0-1.2)	2.2 (2.1-2.3)
PHQ-9 total score ≥ 10 (Moderate to Severe)	TRUE	40.4 (36.6-44.4)	91.4 (91.2-91.5)	1.9 (1.8-2.1)	3.7 (3.4-4.1)
PHQ-9 total score ≥ 11 (Moderate to Severe)	TRUE	36.8 (33.0-40.8)	93.0 (92.9-93.1)	2.2 (2.0-2.4)	4.1 (3.7-4.6)
PHQ-9 total score ≥ 15 (Moderately Severe to Severe)	TRUE	22.6 (19.3-26.0)	97.1 (97.0-97.2)	3.2 (2.8-3.7)	5.7 (4.9-6.5)
PHQ-9 total score ≥ 20 (Severe)	TRUE	7.7 (5.5-10.0)	99.3 (99.2-99.3)	4.4 (3.2-5.7)	5.6 (4.0-7.2)
STB** (Item 9≥ 1, Item 12=Yes, or Item 13=Yes)	TRUE	48.8 (44.8-53.0)	91.9 (91.7-92.0)	2.5 (2.3-2.7)	4.7 (4.3-5.1)

© 2024 Tsui F et al. *JAMA Network Open*.

PHQ-8 total score ≥ 5 and Item 9 ≥ 1	TRUE	31.3 (27.5-35.0)	95.7 (95.6-95.8)	3.0 (2.6-3.3)	5.4 (4.8-6.1)
PHQ-8 total score ≥ 10 and Item 9 ≥ 1	TRUE	25.1 (21.7-28.6)	97.0 (97.0-97.1)	3.5 (3.0-4.0)	**6.1 (5.3-7.0)**
PHQ-8 total score ≥ 5 and (Item 9 ≥ 1, Item 12 = Yes, or Item 13 = Yes)	TRUE	43.0 (39.0-47.2)	93.8 (93.7-94.0)	2.9 (2.6-3.2)	5.4 (4.9-5.9)
PHQ-8 total score ≥ 10 and (Item 9 ≥ 1, Item 12 = Yes, or Item 13 = Yes)	TRUE	30.6 (26.8-34.4)	96.3 (96.2-96.4)	3.4 (3.0-3.8)	**6.1 (5.4-6.9)**

*: F1: a harmonic mean of PPV (precision) and sensitivity (recall), $F1 = 2 \times \frac{precision \times recall}{precision + recall}$.

**: STB: Suicidal Thought and Behavior

Values in boldface indicate the best performance across items within an evaluation metric, e.g., sensitivity.

eTable 3. Prediction Performance of PHQ-9 Item 9 at 4 Prediction Horizons Following the PHQ-9M Screening

Prediction Horizon (days)	Item 9 Score	Sensitivity % (95% CI)	Specificity % (95% CI)	PPV % (95% CI)	F1 % (95% CI)
30	≥1	**47.5 (35.6-61.0)**	95.3 (95.2-95.4)	0.5 (0.3-0.6)	0.9 (0.7-1.2)
	≥2	23.7 (13.6-35.6)	98.7 (98.7-98.8)	0.8 (0.5-1.3)	1.6 (0.9-2.4)
	=3	13.6 (5.1-22.1)	**99.6 (99.5-99.6)**	1.4 (0.6-2.4)	2.6 (1.0-4.3)
90	≥1	43.0 (35.4-50.6)	95.3 (95.2-95.4)	1.1 (0.9-1.3)	2.1 (1.8-2.5)
	≥2	18.4 (12.7-24.7)	98.7 (98.7-98.8)	1.7 (1.2-2.3)	3.2 (2.2-4.2)
	=3	6.3 (2.5-10.1)	**99.6 (99.5-99.6)**	1.8 (0.7-2.9)	2.8 (1.1-4.5)
180	≥1	38.4 (33.0-43.9)	95.3 (95.2-95.4)	1.8 (1.6-2.1)	3.5 (3.0-4.0)
	≥2	16.0 (12.2-20.4)	98.7 (98.7-98.8)	2.8 (2.1-3.6)	4.7 (3.6-6.1)
	=3	7.1 (4.4-10.5)	**99.6 (99.5-99.6)**	3.6 (2.2-5.3)	4.8 (3.0-7.0)
365	≥1	32.1 (28.2-35.7)	95.3 (95.2-95.4)	2.8 (2.5-3.1)	5.2 (4.5-5.7)
	≥2	12.2 (9.5-15.1)	98.7 (98.7-98.8)	3.9 (3.1-4.9)	**5.9 (4.6-7.3)**
	=3	5.1 (3.5-7.1)	**99.6 (99.5-99.6)**	**4.8 (3.2-6.6)**	4.9 (3.3-6.8)

PHQ-9: Patient health questionnaire. PHQ-9M: Patient health questionnaire modified for teens. Values in boldface indicate the best performance across prediction horizons within an evaluation metric, e.g., sensitivity.

eTable 4. Prediction Performance of PHQ-9M Item 12 at 4 Prediction Horizons Following the PHQ-9M Screening

Prediction Horizon (days)	Item 12 Value	Sensitivity % (95% CI)	Specificity % (95% CI)	PPV % (95% CI)	F1 % (95% CI)
30	Yes	**33.9 (22.0-45.8)**	**97.3 (97.2-97.4)**	0.6 (0.4-0.8)	1.1 (0.7-1.5)
90	Yes	29.1 (22.2-36.1)	**97.3 (97.2-97.4)**	1.3 (1.0-1.6)	2.5 (1.9-3.1)

© 2024 Tsui F et al. *JAMA Network Open*.

180	Yes	25.5 (20.7-30.6)	**97.3 (97.2-97.4)**	2.1 (1.7-2.5)	3.9 (3.2-4.7)
365	Yes	22.0 (18.8-25.5)	**97.3 (97.2-97.4)**	**3.4 (2.9-3.9)**	**5.8 (5.0-6.8)**

PHQ-9M: Patient health questionnaire modified for teens.
Values in boldface indicate the best performance across prediction horizons within an evaluation metric, e.g., sensitivity.

eTable 5. Prediction Performance of PHQ-9M Item 13 at 4 Prediction Horizons Following the PHQ-9M Screening

Prediction Horizon (days)	Item 13 Value	Sensitivity (%)	Specificity (%)	PPV (%)	F1 (%)
30	Yes	**40.7 (28.8-52.5)**	**96.0 (95.9-96.1)**	0.5 (0.3-0.6)	0.9 (0.6-1.2)
90	Yes	38.0 (30.4-45.6)	**96.0 (95.9-96.1)**	1.1 (0.9-1.4)	2.2 (1.8-2.6)
180	Yes	35.4 (30.3-40.8)	**96.0 (95.9-96.1)**	1.9 (1.7-2.2)	3.7 (3.1-4.3)
365	Yes	28.8 (25.1-32.6)	**96.0 (95.9-96.1)**	**2.9 (2.6-3.3)**	**5.3 (4.7-6.0)**

PHQ-9M: Patient health questionnaire modified for teens.
Values in boldface indicate the best performance across prediction horizons within an evaluation metric, e.g., sensitivity.

eTable 6. Sensitivity Analysis of 3 Parsimonious Models in 4 Prediction Horizons Following PHQ-9M Screening

Prediction Horizon	CR-3	CR-4	CR-5
**30-Day AUROC (95% CI)**	0.84 (0.78-0.90)	0.86 (0.81-0.92)	**0.88 (0.83-0.93)**
**AUPRC (95% CI)**	0.01 (0.00-0.01)	**0.01 (0.00-0.02)**	**0.01 (0.00-0.02)**
**90-Day AUROC (95% CI)**	0.82 (0.78-0.86)	0.83 (0.79-0.87)	**0.83 (0.80-0.87)**
**AUPRC (95% CI)**	0.01 (0.01-0.02)	0.01 (0.01-0.02)	0.01 (0.01-0.02)
**180-Day AUROC (95% CI)**	0.82 (0.79-0.84)	0.82 (0.80-0.85)	**0.83 (0.80-0.85)**
**AUPRC (95% CI)**	0.02 (0.01-0.02)	**0.02 (0.02-0.03)**	**0.02 (0.02-0.03)**
**365-Day AUROC (95% CI)**	0.79 (0.76-0.81)	**0.79 (0.77-0.81)**	**0.79 (0.77-0.81)**
**AUPRC (95% CI)**	0.02 (0.02-0.03)	**0.03 (0.02-0.03)**	**0.03 (0.02-0.03)**

CR-3/4/5: logistic regression model built from top 3/4/5 variables from 13 PHQ-9M items and PHQ-9 total score; the top n variables were selected from the training data fold. Values in boldface indicate the best performance across prediction horizons within a model, e.g., CR-3.

eTable 7. Confidence Intervals of Performance Differences Between Predictive Models in 4 Prediction Horizons Following PHQ-9M Screening

**Model 1**	**Model 2**	**Horizon (days)**	**AUROC difference (95% Confidence Interval) [Model 1 – Model 2)**	**AUPRC difference (95% Confidence Interval) [Model 1 – Model 2)**
CR-13	CR-9	30	0.0214 (-0.0076 to 0.0516)	0.0023 (-0.0000 to 0.0080)

© 2024 Tsui F et al. *JAMA Network Open*.

CR-13	CR-3	30	0.0413 (0.0135 to 0.0744)*	0.0009 (-0.0023 to 0.0047)
CR-13	PHQ-9 TS	30	0.0137 (-0.0234 to 0.0481)	0.0016 (-0.0018 to 0.0070)
CR-13	CR-9	90	0.0407 (0.0205 to 0.0604)*	0.0034 (0.0003 to 0.0077)*
CR-13	CR-3	90	0.0224 (0.0059 to 0.0393)*	0.0024 (0.0004 to 0.0058)*
CR-13	PHQ-9 TS	90	0.0364 (0.0148 to 0.0565)*	0.0044 (0.0012 to 0.0090)*
CR-13	CR-9	180	0.0388 (0.0230 to 0.0561)*	0.0056 (0.0022 to 0.0099)*
CR-13	CR-3	180	0.0119 (0.0005 to 0.0232)*	0.0029 (0.0009 to 0.0056)*
CR-13	PHQ-9 TS	180	0.0349 (0.0171 to 0.0541)*	0.0063 (0.0022 to 0.0107)*
CR-13	CR-9	365	0.0310 (0.0196 to 0.0428)*	0.0062 (0.0029 to 0.0100)*
CR-13	CR-3	365	0.0144 (0.0054 to 0.0237)*	0.0038 (0.0018 to 0.0061)*
CR-13	PHQ-9 TS	365	0.0248 (0.0112 to 0.0374)*	0.0073 (0.0035 to 0.0115)*

*Non-overlapping 95% confidence intervals

CR-13: logistic regression model built from all 13 individual PHQ-9M items; CR-9: logistic regression model built from all 9 individual PHQ-9 items; CR-3: logistic regression model built from top 3 variables from 13 PHQ-9M items; PHQ-9 TS: PHQ-9 total score. PHQ-9: Patient health questionnaire; PHQ-9M: Patient health questionnaire modified for teens.

© 2024 Tsui F et al. *JAMA Network Open*.

eFigure 1. Boxplots of Days to the First (Closest) Suicide Attempt After the PHQ-9M Screening in Different Subgroups Based on Race, Sex, and Age

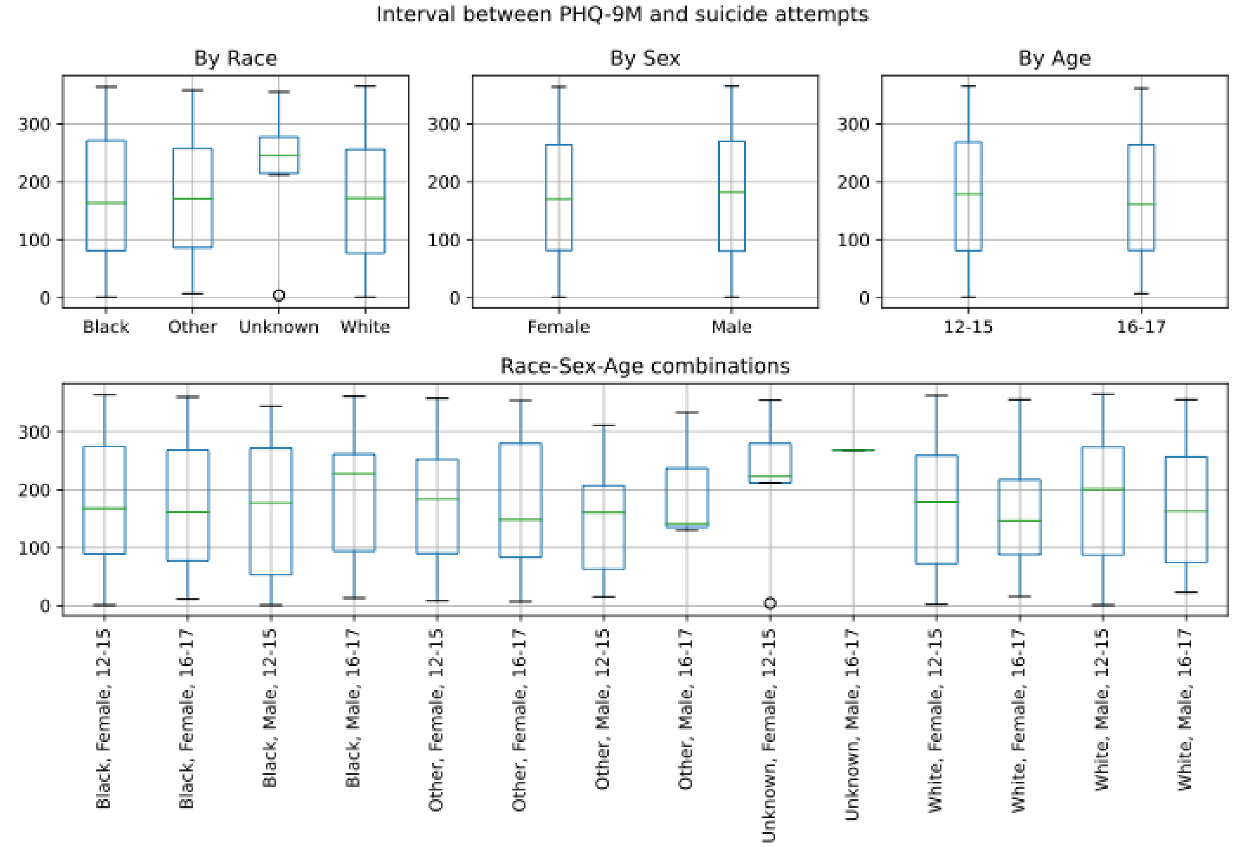

12-15: Age subgroup between 12 and 15 years; 16-17: age subgroup between 16 and 17 years. Note that there were no suicide attempters in the two race-sex-age categories: ‘unknown race, female and age 16-17’ and ‘unknown race, male and age 12-15’. PHQ-9M: Patient health questionnaire modified for teens.

© 2024 Tsui F et al. *JAMA Network Open*.

eFigure 2. A Cumulative Plot With Counts of Suicide Attempts (n = 549) by Days After PHQ-9M Screening

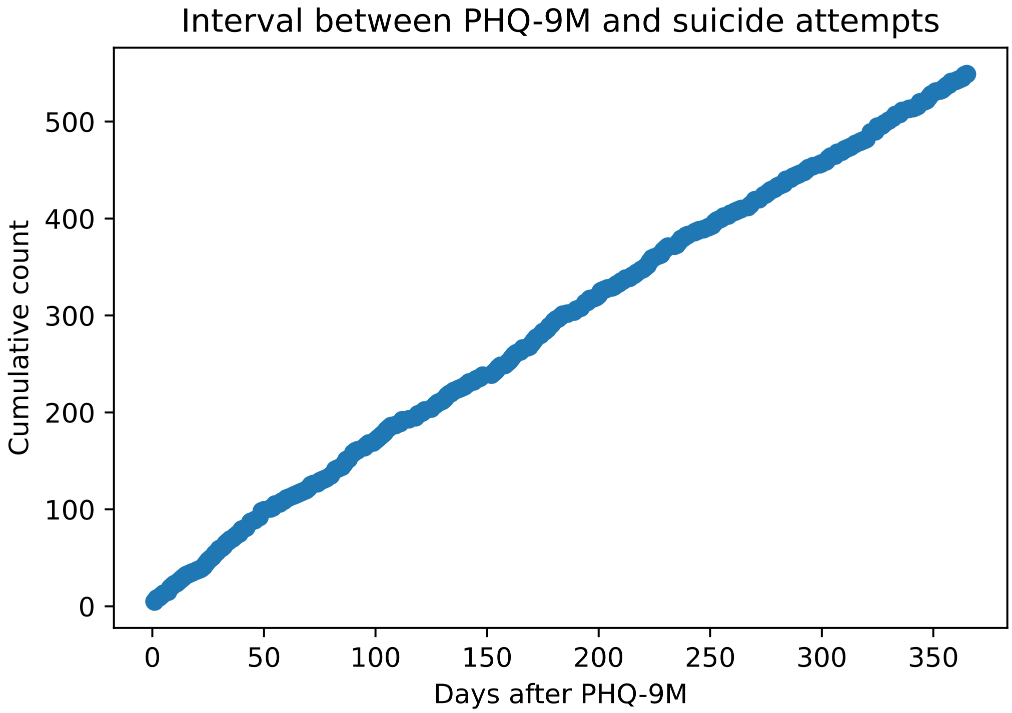

PHQ-9M: Patient health questionnaire modified for teens.

© 2024 Tsui F et al. *JAMA Network Open*.

eFigure 3. Distribution of Races Across 5 Depression Severity Groups Based on PHQ-9 Total Scores

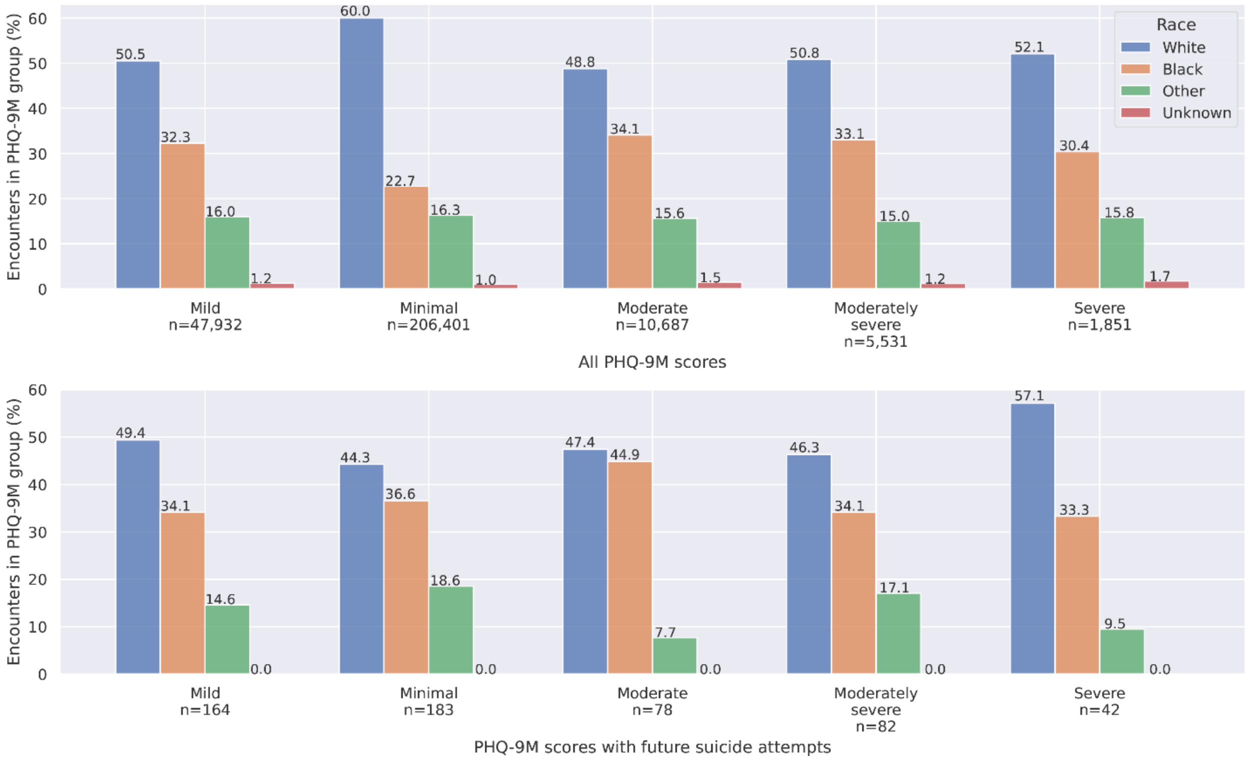

The top figure shows the race distribution among all PHQ-9M respondents. The bottom figure shows the race distribution among PHQ-9M respondents who had a suicide attempt within one year after the screening. 63.2% (347 out of 549) screenings with subsequent suicide attempts had mild or minimal level of PHQ-9 total score. Mild: 0-4; Minimal risk: 5-10; Moderate risk: 11-14; Moderately risk: 15-19; Severe risk: 20-27. PHQ-9: Patient health questionnaire; PHQ-9M: Patient health questionnaire modified for teens.

© 2024 Tsui F et al. *JAMA Network Open*.

eFigure 4. Distributions of PHQ-9 Total Score Severity Groups in the Final Cohort and Screenings With Positive Item 13 (Self-Reported Suicide Attempt History)

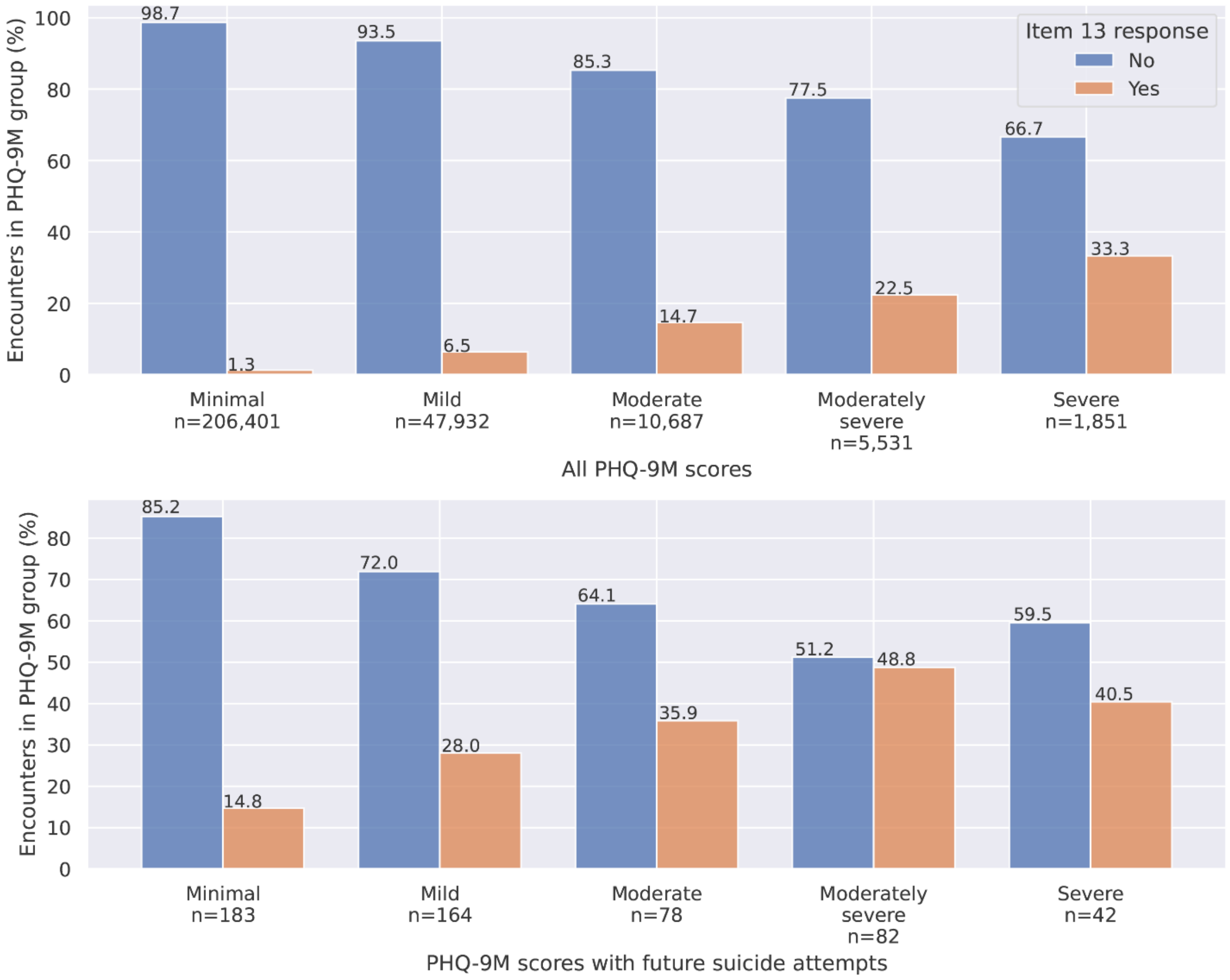

The top figure shows the suicide attempt history distribution among all PHQ-9M respondents. The bottom figure shows the suicide attempt history distribution among PHQ-9M respondents who had a suicide attempt within one year after answering the questionnaire; 71.2% (391 out of 549) screenings with subsequent suicide attempts reported no history of previous suicide attempts in item 13. Minimal depression: 0-4; Mild depression: 5-10; Moderate depression: 11-14; Moderately severe depression: 15-19; Severe depression: 20-27. PHQ-9: Patient health questionnaire; PHQ-9M: Patient health questionnaire modified for teens.

© 2024 Tsui F et al. *JAMA Network Open*.

eFigure 5. Distribution of Item 9 Scores (Self-Reported Suicide Ideation) Across 5 PHQ-9 Severity Groups

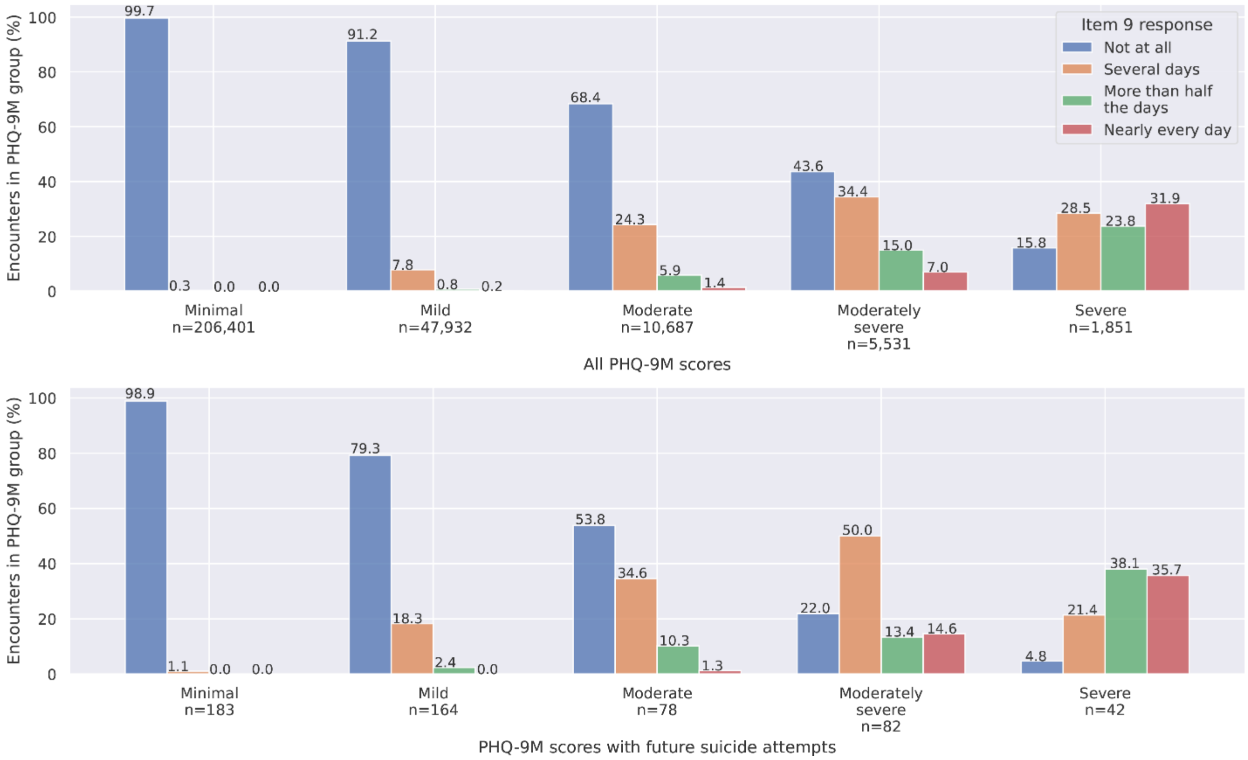

The top figure shows the suicide ideation distribution among all PHQ-9M respondents. The bottom figure shows the suicide ideation distribution among PHQ-9M respondents who had a suicide attempt within one year after answering the questionnaire; 67.9% (373 out of 549) screenings with subsequent suicide attempts reported no suicide ideation (“not at all” option) in item 9. Minimal depression: 0-4; Mild depression: 5-10; Moderate depression: 11-14; Moderately severe depression: 15-19; Severe depression: 20-27. PHQ-9: Patient health questionnaire; PHQ-9M: Patient health questionnaire modified for teens.

© 2024 Tsui F et al. *JAMA Network Open*.

eFigure 6. Pairwise Spearman Correlation Matrix of Individual PHQ-9M Items

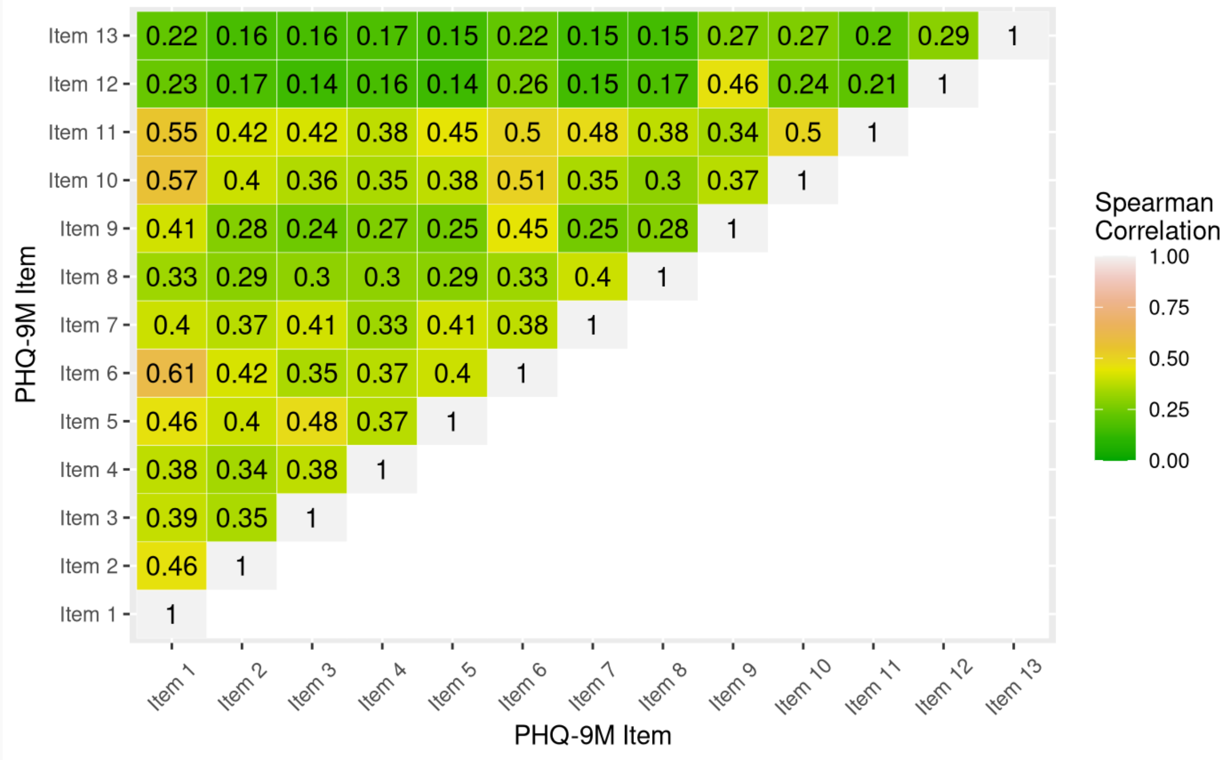

PHQ-9M: Patient health questionnaire modified for teens.

© 2024 Tsui F et al. *JAMA Network Open*.

eFigure 7. Ranked Unadjusted Hazard Ratios (uHRs) for All 13 Items in the PHQ-9M Questionnaire and the Severity of the PHQ-9 Total Score

Variable	Hazard ratio (95% CI)
PHQ-9 score: Severe	28.21 (20.17-39.46)
PHQ-9 score: Moderately severe	17.74 (13.67-23.03)
Item 13	12.03 (10-14.47)
Item 12	10.99 (8.98-13.45)
Item 9	9.77 (8.16-11.69)
Item 10	9.05 (7.56-10.82)
PHQ-9 score: Moderate	8.49 (6.51-11.07)
Item 1	6.55 (5.51-7.79)
Item 6	5.87 (4.97-6.95)
Item 11	5.66 (4.77-6.72)
Item 4	4.23 (3.57-5)
Item 2	4.16 (3.51-4.94)
Item 5	4.13 (3.42-4.98)
PHQ-9 score: mild	3.88 (3.14-4.79)
Item 3	3.7 (3.09-4.43)
Item 8	3.66 (3.06-4.37)
Item 7	3.46 (2.91-4.1)

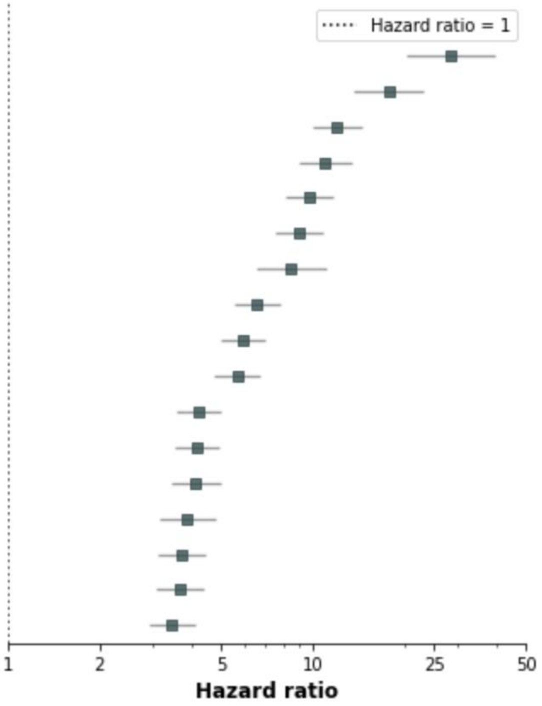

The uHRs for questions 1 to 13 had a binary value (‘0’ or ‘no’ vs. ‘≥1’ or ‘yes’) with a reference group of ‘0’ or ‘no’, and the uHRs for score severity had the ‘Minimal’ (score < 5) severity as a reference group. Mild: PHQ-9 total score (TS) between 5 and 10; Moderate: PHQ-9 TS between 11 and 14; Moderately Severe: PHQ-9 TS between 15 and 19; Severe: PHQ-9 TS between 20 and 27. Item 1: Feeling down, depressed, irritable, or hopeless? Item 2: Little interest or pleasure in doing things? Item 3: Trouble falling asleep, staying asleep, or sleeping too much? Item 4: Poor appetite, weight loss, or overeating? Item 5: Feeling tired, or having little energy? Item 6: Feeling bad about yourself — or feeling that you are a failure, or that you have let yourself or your family down? Item 7: Trouble concentrating on things like school work, reading, or watching TV? Item 8: Moving or speaking so slowly that other people could have noticed? Item 9: Thoughts that you would be better off dead, or of hurting yourself in some way? Item 10: (supplemental item): In the past year have you felt depressed or sad most days, even if you felt okay sometimes? Item 11: (supplemental item) If you are experiencing any of the problems on this form, how difficult have these problems made it for you to do your work, take care of things at home or get along with other people? Item 12: (supplemental item) Has there been a time in the past month when you have had serious thoughts about ending your life? Item 13: (supplemental item) Have you EVER, in your WHOLE LIFE, tried to kill yourself or made a suicide attempt?
PHQ-9: Patient health questionnaire; PHQ-9M: Patient health questionnaire modified for teens.

© 2024 Tsui F et al. *JAMA Network Open*.

eFigure 8. Precision Recall Curves of 5 Predictors Derived From the PHQ-9M Questionnaire for the Prediction of Subsequent Suicide Attempts

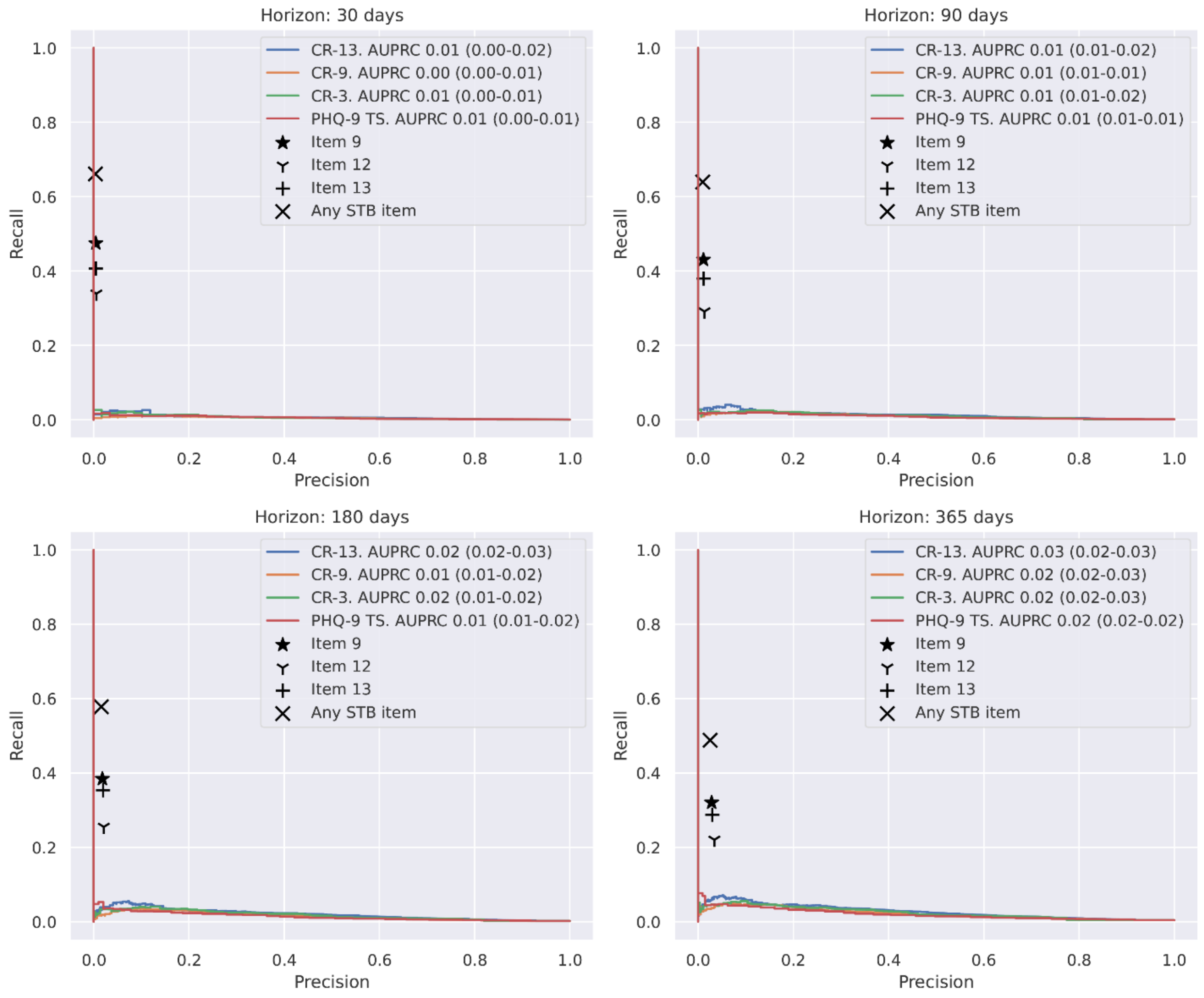

PHQ-9: Patient health questionnaire; PHQ-9M: Patient health questionnaire modified for teens; *PHQ-9 TS*: the PHQ-9 total score; *CR-9*: Cox regression model from the core 9 items in the PHQ-9M questionnaire and score severity; *CR-13*: Cox regression model from all 13 items in the PHQ-9M questionnaire and score severity. CR-3: Cox regression using the top-3 most predictive items among the 13 items of the PHQ-9M questionnaire and score severity; *Any STB item*: binary defined as endorsement of any of the three suicide thoughts or behavior items is positive (item 9 ≥1, item 12=yes, or item 13=yes). Four prediction horizons following PHQ-9M questionnaires were evaluated. The control group is the same across horizons (n=129,479). The suicide attempt group size varies across horizons as follows: 30 days (n=59, top left), 90 days (n=158, top right), 180 days (n=294, bottom left), and 365 days (n=549, bottom right).

© 2024 Tsui F et al. *JAMA Network Open*.

eReferences.

1. Bell CC. DSM-IV: Diagnostic and Statistical Manual of Mental Disorders. JAMA. 1994;272(10):828–829. doi:10.1001/jama.1994.03520100096046
2. Manea L, Gilbody S, McMillan D. A diagnostic meta-analysis of the Patient Health Questionnaire-9 (PHQ-9) algorithm scoring method as a screen for depression. Gen Hosp Psychiatry. 2015 Jan-Feb;37(1):67-75. doi: 10.1016/j.genhosppsych.2014.09.009. Epub 2014 Sep 23. PMID: 25439733.
3. Richardson LP, McCauley E, Grossman DC, McCarty CA, Richards J, Russo JE, Rockhill C, Katon W. Evaluation of the Patient Health Questionnaire-9 Item for detecting major depression among adolescents. Pediatrics. 2010 Dec;126(6):1117-23. doi: 10.1542/peds.2010-0852. Epub 2010 Nov 1. PMID: 21041282; PMCID: PMC3217785.
4. Johnson JG, Harris ES, Spitzer RL, Williams JB. The patient health questionnaire for adolescents: validation of an instrument for the assessment of mental disorders among adolescent primary care patients. J Adolesc Health. 2002 Mar;30(3):196-204. doi: 10.1016/s1054-139x(01)00333-0. PMID: 11869927.

© 2024 Tsui F et al. *JAMA Network Open*.
