# Supplementary material for: Risk for Suicide Attempts Assessed Using the Patient Health Questionnaire–9 Modified for Teens
Source: JAMA Netw Open. 2024 Oct 8;7(10):e2438144. doi: 10.1001/jamanetworkopen.2024.38144 (PMC11581555; doi:10.1001/jamanetworkopen.2024.38144)
Supplement: Supplement 2. — Data Sharing Statement [file jamanetwopen-e2438144-s002.pdf]

Data Sharing Statement

Tsui. Risk for Suicide Attempts Assessed Using the Patient Health Questionnaire–9 Modified for Teens. *JAMA Netw Open*. Published October 08, 2024.
doi:10.1001/jamanetworkopen.2024.38144

Data
Data available: No
